# Neonatal Mortality and Its Associated Factors Among Neonates in Ethiopia: A Systematic Review and Meta‐Analysis

**DOI:** 10.1002/puh2.70332

**Published:** 2026-08-10

**Authors:** Temesgen Gebeyehu Wondmeneh, Oumer Abdulkadir Ebrahim

**Affiliations:** ^1^ Department of Public Health College of Medical and Health Science Samara University Semera Ethiopia

**Keywords:** Ethiopia, mortality, neonates

## Abstract

**Background:**

Despite global declines in neonatal mortality, Sub‐Saharan Africa continues to bear a disproportionate burden. In Ethiopia, available evidence is largely derived from facility‐based studies, yet pooled evidence from these studies remains limited. Therefore, this study aimed to estimate the pooled proportion of neonatal deaths among admitted neonates and identify associated factors in Ethiopia.

**Methods:**

We searched major databases, including Web of Science, Scopus, PubMed, Google Scholar, and African Journals Online. Data were extracted using standardized Microsoft Excel spreadsheet. Study quality was assessed using the Joanna Briggs Institute appraisal checklist. A random‐effects meta‐analysis estimated pooled proportion of neonatal deaths, with heterogeneity measured by *I* ^2^ and publication bias assessed using a funnel plot and Egger's test.

**Results:**

The pooled proportion of admitted neonatal deaths in Ethiopia was 14.31% (95% confidence interval [CI]: 12.59–16.03), indicating a high burden of neonatal deaths within healthcare facilities, with substantial heterogeneity across studies (*I* ^2^ = 98.93%). Given this extreme heterogeneity, the pooled estimate should be interpreted cautiously and should not be considered a fixed national figure. Lack of antenatal care (ANC) (adjusted odds ratio [AOR] = 2.7, 95% CI: 1.5–3.96), birth asphyxia (AOR = 5.1, 95% CI: 3.0–7.1), low‐birth weight (AOR = 4.1, 95% CI: 1.6–6.6), preterm birth (AOR = 2.2, 95% CI: 1.3–3.1), and hypothermia (AOR = 2.4, 95% CI: 1.2–3.7) were identified as significant risk factors for neonatal deaths.

**Conclusion:**

A considerable proportion of neonatal deaths occurred among admitted neonates in Ethiopia, indicating a high burden of neonatal mortality within healthcare facilities. The major risk factors for neonatal deaths were lack of ANC, asphyxia, low‐birth weight, preterm birth, and hypothermia. As the evidence was derived predominantly from facility‐based studies, the findings mainly reflect the proportion of deaths among neonates receiving care in health facilities and should not be considered nationally representative of all neonates in Ethiopia.

## Graphical abstract

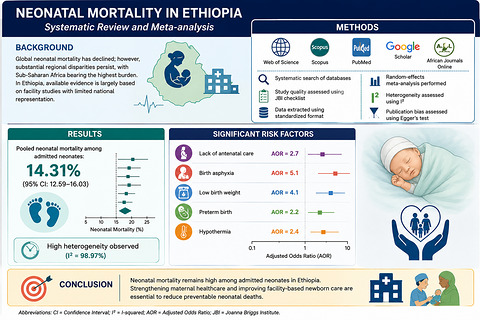

## 1 Background

The first 28 days of life, the neonatal period, are the most vulnerable time for a child's survival. Children face the highest risk of death during the first month of life, with an average global rate of 18 deaths per 1000 live births in 2021 [1]. In low‐ and middle‐income countries, the neonatal mortality rate was 28.7 per 1000 live births [2]. Sub‐Saharan Africa reported the highest neonatal mortality rate in 2020 at 27 deaths per 1000 live births, followed by Central and Southern Asia with 23 deaths per 1000 live births [3]. A Demographic and Health Survey (DHS) study conducted in East Africa reported a neonatal mortality rate of 33.6 per 1000 live births [4]. In Ethiopia, neonatal mortality ranged from 20.7 to 29.1 per 1000 live births [5, 6, 7, 8]. In the emerging regions of Ethiopia, the neonatal mortality rate was 34.9 per 1000 live births [9]. A study conducted in eastern Ethiopia reported a neonatal mortality rate of 20% [10]. The prevalence of early neonatal mortality in Ethiopia ranged from 36 [11] to 41.8 per 1000 live births [12], whereas preterm neonatal mortality was approximately 29% [13, 14].

The risk factors for neonatal death in Ethiopia include maternal age, male sex, delayed initiation of breastfeeding, lack of antenatal care (ANC) follow‐up, nonuse of iron and folic acid supplementation, the absence of postnatal care visits, low‐birth weight, extreme prematurity, HIV infection, and maternal near‐miss events [15, 16, 17]. Age of the mother below 20 years, home delivery, pregnancy‐related complications, referred neonates, and preterm birth were risk factors for neonatal mortality [18, 19]. Neonatal death was caused by small gestational age, respiratory distress syndrome, hypothermia, sepsis, jaundice, and prenatal asphyxia [20, 21].

Previous studies on neonatal mortality in Ethiopia have been limited to specific geographical areas, with widely varying estimates that limit their usefulness for informing practice and policy. National analyses based on Ethiopian DHS (EDHS) data lack key clinical variables and are subject to recall bias. Most studies included in the current analysis are derived from health facility settings, which are important for capturing clinical variables and improving healthcare service management. However, these estimates mainly reflect proportion of deaths among admitted neonates rather than population‐level neonatal mortality rates. Therefore, this study synthesizes evidence from multiple district‐level primary studies to provide an updated estimate of neonatal deaths among facility‐based populations in Ethiopia and to identify its associated factors.

## 2 Methods

### 2.1 Protocol Registration and Reporting

The current systematic review and meta‐analysis protocol was registered in the International Prospective Register of Systematic Reviews (PROSPERO) (ID: CRD42024513474). The Preferred Reporting Items for Systematic Reviews and Meta‐Analyses (PRISMA) guidelines [22] were used to report this meta‐analysis (Supporting Information S1).

### 2.2 Search Strategy

A comprehensive and systematic literature search was conducted in Web of Science, Scopus, PubMed, African Journals Online, and Google Scholar to identify relevant studies. To minimize the risk of missing unpublished or grey literature, additional searches were performed in Ethiopian higher education institutional repositories. A comprehensive search strategy was developed using both Medical Subject Headings (MeSH) and free‐text terms. The search included a wide range of synonyms and related epidemiological terms to improve sensitivity, such as: (“neonatal mortality” OR “neonatal death” OR “newborn death” OR “neonatal survival” OR “infant mortality”) AND (“Ethiopia”). Boolean operators (“AND” and “OR”) were appropriately applied to combine search terms and synonyms. Studies published from 2016 onward were included because 2016 marked the release of the most recent nationally representative EDHS, which coincided with substantial changes in maternal and newborn health policies and service delivery in Ethiopia. Restricting inclusion to studies published from 2016 onward was intended to ensure that the synthesized evidence reflects contemporary clinical, epidemiological, and health‐system contexts while minimizing the influence of outdated estimates. This search, which was guided by population (neonates), condition (mortality), and context (Ethiopia) (PCCO framework), was initially conducted between March 1, 2024, and March 10, 2024, and subsequently updated from January 25 to January 30, 2025, and from March 28 to March 31, 2026 (Supporting Information S2).

### 2.3 Study Selection

Using predefined search terms and database filters, initial search results were retrieved. All identified articles were exported to EndNote X8.1 software, and duplicates were removed. The remaining articles were screened by title and abstract according to the objective of this systematic review. Full‐text articles deemed relevant were then retrieved. T.G.W. and O.A.E. independently assessed the full‐text articles for eligibility, and any disagreements were resolved through discussion and consensus. Following full‐text assessment, eligible studies were included in the final meta‐analysis.

### 2.4 Inclusion and Exclusion Criteria

Observational studies (i.e., cross‐sectional, case‐control, and cohort) conducted in Ethiopia that reported the magnitude of neonatal deaths and its associated factors were included. Articles published in English were also considered. Studies published between 2016 and 2026 were included. When the same study was published in different journals, only one version was included. Studies that did not report the outcome of interest (neonatal deaths) were excluded. Studies involving specific populations, such as early neonates, preterm neonates, asphyxiated neonates, neonates with sepsis, and low‐birth‐weight neonates, were excluded due to their limited representativeness and the potential overestimation of mortality. Articles lacking full‐text access were excluded after contacting the corresponding authors by email at least twice. Review articles, commentaries, editorials, EDHS reports, and national‐level studies were also excluded.

### 2.5 Operational Definition

Neonate: The neonatal period is defined as the first 28 days of life [23].

Neonatal deaths: Neonatal death refers to the death of a live‐born infant within the first 28 days of life. The numerator for neonatal mortality is the number of deaths among infants aged less than 28 days during a specified period, whereas the denominator is the total number of live births reported during the same period [24].

### 2.6 Outcome Measurement

The main outcome of interest was the proportion of neonatal deaths among neonates included in the primary studies in Ethiopia, which is reported in the primary article either as a percentage or as a frequency. It was computed by dividing the number of neonates who died within the first 28 days of neonatal life by the total number of live births (sample size) multiplied by 100.

### 2.7 Data Extraction

Two authors independently extracted data from the included studies. Disagreements between the two authors were resolved through scientific consensus. Using a standardized data extraction form developed in Microsoft Excel, the following data were extracted: first author's name, publication date, study population, study region, study design, sample size, and number of neonatal deaths. Details are provided in Supporting Information S3.

### 2.8 Quality Assessment

The quality of each included study was independently appraised by two authors (T.G.W. and O.A.E.) using the Joanna Briggs Institute (JBI) Critical Appraisal Checklist for analytical cross‐sectional studies [25]. The tool includes four response options: Yes, No, Unknown, and Not Applicable; a score of 1 was assigned for “Yes” and 0 for all other responses. The scores were summed and converted to percentages. Studies with a quality score of 60% or higher were included in this meta‐analysis. During the critical appraisal process, disagreements between the two authors were resolved through scientific consensus.

### 2.9 Data Synthesis and Statistical Analysis

The extracted data were entered into a Microsoft Excel spreadsheet and then imported into Stata version 17 for analysis. The primary outcome (neonatal deaths) was pooled using a random‐effects meta‐analysis model to account for expected heterogeneity across studies [26]. Heterogeneity was assessed using Cochran's *Q* test and the *I* ^2^ statistic, with values of 25%, 50%, and 75% indicating low, moderate, and high heterogeneity, respectively [27]. Publication bias was evaluated using visual inspection of funnel plots and Egger's regression test [28, 29]. As publication bias was detected, a trim‐and‐fill analysis was subsequently conducted. Sensitivity analysis was performed to assess the influence of individual studies on the overall pooled estimates [30]. In addition, subgroup analyses were conducted on the basis of region, study design, study population, publication year, and sample size. Furthermore, meta‐regression analyses based on publication year and sample size were conducted to further explore sources of heterogeneity. For the analysis of associated factors, effect sizes were extracted from the included studies. Whenever available, only adjusted odds ratios (AORs) along with their corresponding 95% confidence intervals (CIs) were pooled to minimize confounding. Meta‐analyses for each determinant were conducted only when at least three studies reported effect estimates to ensure adequate statistical power, and the number of studies included for each factor was documented. To ensure comparability across variables, the operational definitions of each variable were reviewed across studies, and efforts were made to harmonize them according to standard criteria. Any remaining inconsistencies were considered during the interpretation of the findings. A random‐effects model was used to pool all effect estimates for the associated factors due to anticipated variability among the studies. Finally, the results were presented using forest plots, tables, and narrative summaries.

## 3 Results

### 3.1 Search Results

**FIGURE 1:**
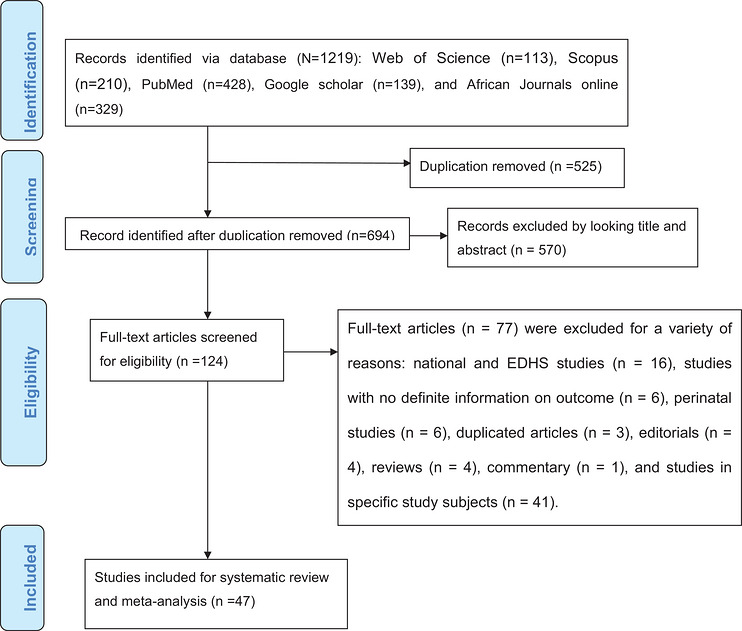
The figure shows PRISMA flow diagram that shows the selection of articles for systematic review and meta‐analysis. EDHS, Ethiopian DHS.

Advanced search strategies across multiple databases yielded a total of 1219 articles. Specifically, Web of Science, Scopus, and PubMed provided 113, 210, and 428 articles, respectively. Additionally, 139 and 329 articles were retrieved from Google Scholar and African Journals Online, respectively. A total of 525 duplicate articles were removed using EndNote X8.1. On the basis of title and abstract screening, 570 irrelevant articles were excluded. Following full‐text assessment, 77 articles were excluded for various reasons: A total of 11 studies used EDHS data; 6 lacked sufficient information; 6 focused on perinatal outcomes; 5 were conducted at the national level; 3 were duplicate publications (the same results published in different journals); 4 were review articles; 1 was a commentary; and 41 focused on specific subgroups (e.g., early, preterm, asphyxiated, low‐birth‐weight, and septic neonates). Finally, 47 eligible studies were identified and included in the meta‐analysis (Figure 1).

### 3.2 Study Characteristics

In this meta‐analysis, a total of 47 studies comprising 52,853 neonates were included. Among these, 5129 neonatal deaths were reported. Of the included studies, 37 were retrospective, and 10 were prospective in design. Regionally, the majority of studies were conducted in the Amhara region (*n* = 15), followed by the Southern Nations, Nationalities, and Peoples’ Region (SNNPR) (*n* = 8). Five studies were conducted in the Oromia region. Addis Ababa and Tigray each contributed four studies, whereas three studies were also conducted in each of the Somali and Sidama regions. Two studies were identified from each of the Dire Dawa City Administration and the Afar region, whereas one study was conducted in the Harari region. The studies were published between 2016 and 2026. Most of the included studies were facility‐based (*n* = 44), whereas the remaining were community‐based. The sample sizes ranged from 225 [31] to 11,306 [32] participants. The reported neonatal deaths varied widely, from 0.96% [33] to 34.2% [34] (Table 1).

**TABLE 1:** Characteristics of the included studies.

ID	References	Region	Study design	Study subjects	Sample size	Cases (%)
1.	A. Deressa et al. [34]	Sidama	Retrospective	Neonates	325	111 (34.2)
2.	A. W. Tadesse et al. [35]	Afar	Retrospective	Neonates	391	57 (14.6)
3.	A. G. Demisse et al. [36]	Amhara	Retrospective	Neonate	769	110 (14.3)
4.	A. E. Farah et al. [37]	Somali	Retrospective	Neonates	792	45 (5.7)
5.	A. Mersha et al. [33]	SNNP	Prospective	Neonates	6769	65 (0.96)
6.	A. Eyeberu et al. [38]	Harar	Retrospective	Neonates	821	118 (14.4)
7.	A. A. Limaso et al. [39]	Sidama	Prospective	Neonates	584	24 (4.1)
8.	A. Yeshaneh et al. [40]	Amhara	Prospective	Neonates	422	129 (30.6)
9.	A. Alebel et al. [41]	Amhara	Prospective	Neonates	513	109 (21.3)
10.	A. M. Dessie et al. [42]	Amhara	Retrospective	Neonates	926	41 (4.4)
11.	A. Kassie et al. [43]	Amhara	Retrospective	Neonates	870	190 (21.8)
12.	B. A. Mengistu et al. [44]	Amhara	Prospective	Neonates	612	114 (18.6)
13.	B. Berhanu et al. [45]	SNNP	Retrospective	Neonates	367	39 (10.6)
14.	B. Chekole et al. [46]	SNNP	Retrospective	Neonates	375	85 (22.7)
15.	E. M. Roro et al. [47]	Oromia	Retrospective	Neonates	2090	183 (8.8)
16.	F. B. Hadgu et al. [48]	Tigray	Retrospective	Neonates	1785	298 (16.7)
17.	G. Thomas et al. [49]	Dire Dawa	Retrospective	Neonates	376	43 (11.4)
18.	G. K. Getahun et al. [50]	Addis Ababa	Retrospective	Neonates	459	14 (3.1)
19.	G. J. Chan et al. [51]	Amhara	Retrospective	Neonates	2411	75 (3.1)
20.	H. A. Mohamed et al. [52]	Somali	Retrospective	Neonates	510	95 (18.6)
21.	H. G. Mengesha et al. [53]	Tigray	Prospective	Neonates	1152	68 (5.9)
22.	K. Taye et al. [31]	Sidama	Retrospective	Neonates	225	32 (14.2)
23.	M. Kokeb et al. [54]	Amhara	Retrospective	Neonates	325	75 (32.1)
24.	M. W. Gebremichael et al. [32]	Tigray	Retrospective	Neonates	11,306	189 (1.67)
25.	M. Z. Ayichew et al. [55]	Addis Ababa	Retrospective	Neonates	570	44 (7.7)
26.	M. O. Osman et al. [56]	Somali	Retrospective	Neonates	385	79 (20.5)
27.	D. Samuel et al. [57]	SNNP	Retrospective	Neonates	332	67 (20.2)
28.	S. K. Demis et al. [58]	Amhara	Retrospective	Neonates	422	52 (12.3)
29.	T. Tolossa et al. [59]	Oromia	Prospective	Neonates	412	63 (15.3)
30.	T. Derese et al. [60]	Dire Dawa	Retrospective	Neonates	499	44 (8.8)
31.	T. Abera et al. [61]	Oromia	Retrospective	Neonates	289	53 (18.34)
32.	T. Mekonnen et al. [62]	SNNP	Retrospective	Neonates	1316	300 (22.8)
33.	T. Tewabe et al. [63]	Amhara	Retrospective	Neonates	391	52 (13.29)
34.	T. T. Orsido et al. [64]	SNNP	Retrospective	Neonates	964	159 (16.5)
35.	M. M. Menalu et al. [65]	Addis Ababa	Retrospective	Neonates	434	48 (11.1)
36.	T. W. Gudayu et al. [66]	Amhara	Retrospective	Neonates	504	87 (17.3)
37.	S. Moges et al. [67]	SNNP	Retrospective	Neonates	1104	207 (18.8)
38.	F. Cavallin et al. [68]	Oromia	Retrospective	Neonates	4182	709 (17)
39.	A. B. Awoke et al. [69]	Amhara	Retrospective	Neonates	323	28 (8.7)
40.	G. T. Simeneh et al. [70]	Addis Ababa	Retrospective	Neonates	313	27 (8.6)
41.	W. Zekarias et al. [71]	Afar	Retrospective	Neonates	479	87 (18.16)
42.	A. S. Enawgaw et al. [72]	Amhara	Retrospective	Neonates	638	217 (34)
43.	B. Adugna et al. [73]	Amhara	Retrospective	Neonates	1598	163 (10.2)
44.	M. Jemal et al. [74]	SNNP	Prospective	Neonates	1080	56 (5.2)
45.	G. D. Feyissa et al. [75]	Oromia	Prospective	Neonates	504	119 (23.6)
46.	G. B. Haile et al. [76]	Tigray	Prospective	Neonates	334	42 (12.6)
47.	N. Fentaw et al. [77]	Amhara	Retrospective	Neonates	604	117 (19.4)

Abbreviations: AN, asphyxia neonate; LBW, low‐birth weight; P, prevalence; SNNP, Southern Nations, Nationalities, and People.

### 3.3 Quality of the Included Studies

The quality of the included studies was assessed independently by T.G.W. and O.A.E. using the JBI Critical Appraisal Checklist for analytical cross‐sectional studies. Most of the included studies were retrospective, whereas a few were prospective. The quality scores ranged from 5 to 8 out of 8. A threshold of 60% or higher was applied to ensure the inclusion of high‐quality studies. The methodological quality of the included studies ranged from 62.5% to 100%. Therefore, all included studies were considered to be of high quality (Supporting Information S4).

### 3.4 Pooled Prevalence of Neonatal Mortality in Ethiopia

**FIGURE 2:**
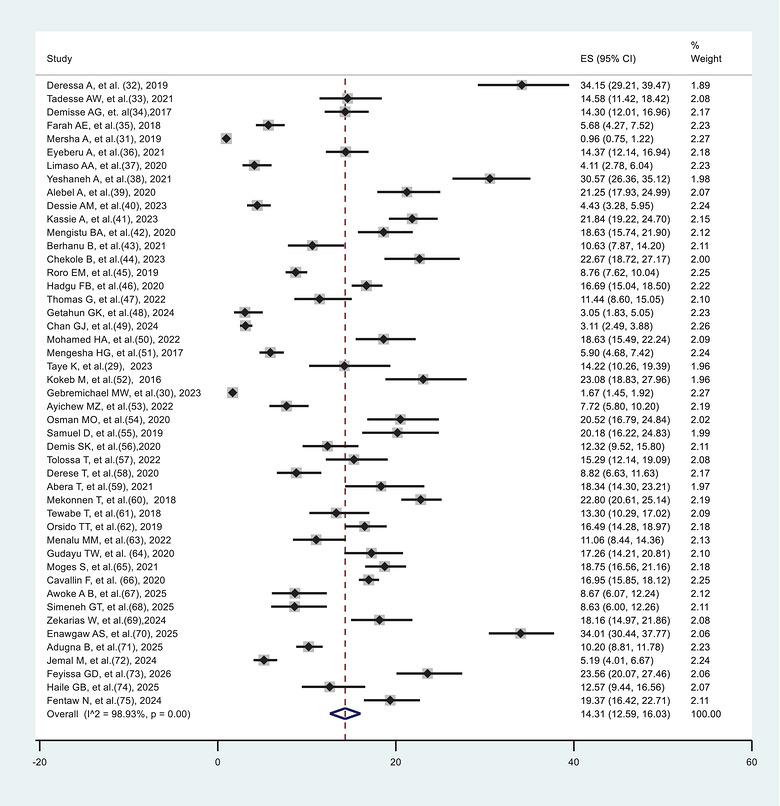
Pooled proportion of neonatal deaths in Ethiopia.

In this systematic review, a total of 52,853 neonates were included, of whom 5129 died. The pooled proportion of admitted neonatal deaths across the included studies was 14.31% (95% CI: 12.59–16.03), largely driven by facility‐based evidence. There was a high level of statistically significant heterogeneity among the included studies (*I* ^2^ = 98.93%, *p* < 0.001) (Figure 2).

### 3.5 Subgroup Analysis

To explore sources of heterogeneity, subgroup analyses were conducted by region, study setting, study design, publication year, and sample size. The highest neonatal deaths were observed in Sidama (17.4%), Amhara (16.7%), Afar (16.4%), and Oromia (16.3%), whereas lower estimates were reported in Addis Ababa (7.5%) and Tigray (9.1%). Similarly, relatively high estimates were noted in SNNP (14.6%), Somalia (14.8%), and Harari (14.4%). Institution‐based studies reported substantially higher neonatal deaths (15.2%; 95% CI: 13–18) compared to community‐based studies (3.3%; 95% CI: 1.2–5.4). Retrospective studies showed a slightly higher estimate (14.6%; 95% CI: 12.1–17.1) than prospective studies (13.5%; 95% CI: 9–18.1). The proportion of neonatal deaths was comparable across publication periods, at 14.8% (95% CI: 9.9–19.7) for studies published between 2016 and 2019 and 14.3% (95% CI: 12.6–16.0) for those published between 2020 and 2025. Similarly, studies with smaller sample sizes (≤421) reported higher deaths (16.3%; 95% CI: 13.3–19.4) compared to those with larger sample sizes (>421) (13.4%; 95% CI: 11.5–15.3). High heterogeneity was observed across most subgroups (Table 2).

**TABLE 2:** Subgroup analysis.

Variables	Categories	Proportion of deaths (95% CI)	*I* ^2^ (%)	*p* value	*df*
Region	Amhara	16.7% (12.3–21.1)	98.4	<0.001	14
	SNNP	14.6% (7.8–21.5)	99.3	<0.001	7
	Addis Ababa	7.5% (3.8–11.2)	90	<0.001	3
	Oromia	16.3% (11.4–21.3)	96.7	<0.001	4
	Tigray	9.1% (2.2–16.1)	99.1	<0.001	3
	Sidama	17.4% (0.34–34.4)	—	—	2
	Somalia	14.8% (4.3–25.4)	—	—	2
	Dire Dawa	9.8% (7.8–11.8)	—	—	1
	Harar	14.4% (12.1–16.9)	—	—	—
	Afar	16.4% (13.9–18.9)	—	—	1
Study setting	Community‐based	3.3% (1.2–5.4)	99	—	2
	Institutional‐based	15.2% (13–18)	98.9	<0.001	43
Study design	Retrospective	14.6% (12.1–17.1)	98.9	<0.001	36
	Prospective	13.5% (9–18.1)	98.8	<0.001	9
Publication year	2016–2019	14.8% (9.9–19.7)	99.1	<0.001	10
	2020–2025	14.3% (12.6–16)	98.8	<0.001	35
sample size	≤421	16.3% (13.3–19.4)	90	<0.001	15
	>421	13.4% (11.5–15.3)	99.1	<0.001	32

Abbreviations: SNNP, Southern Nation, Nationality, and People.

A meta‐regression analysis was performed to identify potential sources of heterogeneity in neonatal death estimates using publication year and sample size as covariates. The overall model was statistically significant (*F* (2, 44) = 3.85, *p* = 0.0288, Knapp–Hartung adjustment), indicating that the included variables jointly explained a significant proportion of the variability across studies. The model accounted for 56.19% of the between‐study variance, suggesting substantial explanatory power. Sample size was found to be a significant predictor of effect size (*β* = −6.80 × 10^−6^, *p* = 0.008), with larger studies reporting lower neonatal death estimates, which may reflect small‐study effects or differences in study precision and representativeness. In contrast, publication year was not significantly associated with neonatal deaths (*β* = 0.00029, *p* = 0.947), indicating no evidence of a temporal trend in the reported estimates. After adjusting for these covariates, the residual heterogeneity was negligible (*I* ^2^ = 0.00%; *τ* ^2^ = 0.000281), suggesting that most of the observed heterogeneity was explained by the variables included in the model (Table 3).

**TABLE 3:** Meta‐regression based on sample size and publication years.

Effect size	Coefficient	95% CI		*p* value
		Lower	Upper	
Publication years	0.0002889	−0.008349	0.0089268	0.947
Sample size	−6.80e−06	−0.0000118	−1.84e−06	0.008

### 3.6 Publication Bias

**FIGURE 3:**
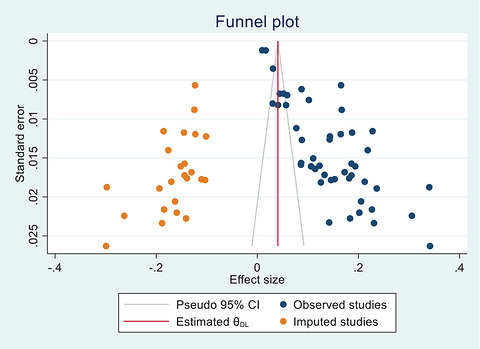
Funnel plot to examine publication bias.

Publication bias was assessed using a funnel plot and Egger's test. The funnel plot indicated asymmetry among the included studies, and Egger's test confirmed the presence of publication bias (bias = 10.4, 95% CI: 8.7–12.1; *p* < 0.001). Therefore, a trim and fill meta‐analysis was performed. The analysis imputed 24 potentially missing studies, yielding a total of 71 studies after combining observed and imputed data. Following imputation, the pooled proportion of neonatal deaths decreased from 14.31% (95% CI: 12.59–16.03) to 4.1% (95% CI: 2.3–5.9), representing a reduction of 10.2%. The trim and fill method produced a more symmetrical funnel plot by imputing the missing studies on the left side of the distribution (Figure 3).

### 3.7 Sensitivity Analysis

**FIGURE 4:**
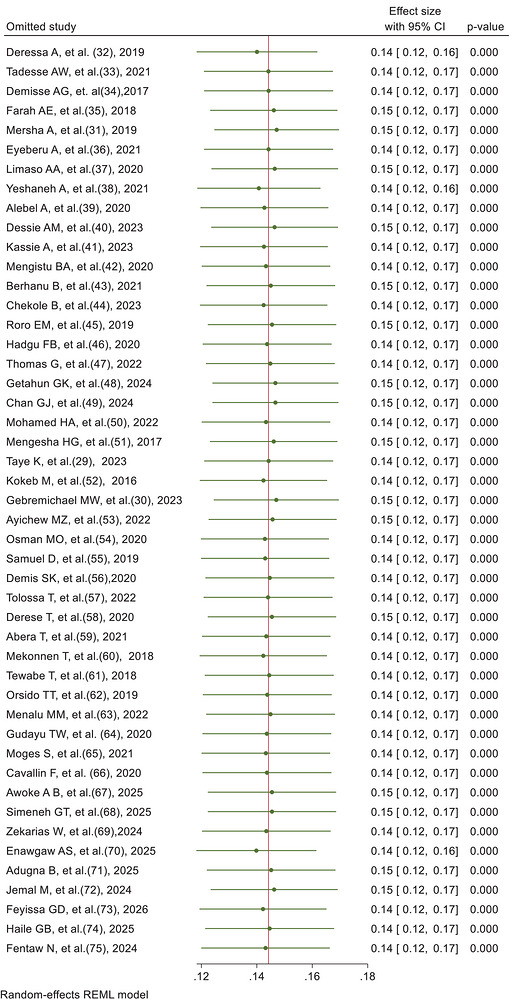
Sensitivity analysis for neonatal deaths.

The leave‐one‐out sensitivity analysis using the random‐effects model showed that the pooled proportion of neonatal deaths remained largely unchanged when each study was omitted in turn. The estimated effect sizes consistently ranged between approximately 0.14 and 0.15, with overlapping CIs across all iterations. This indicates that no single study had a disproportionate influence on the overall pooled estimate. Therefore, the findings of the meta‐analysis are robust, and the results of the sensitivity analysis confirm the stability and reliability of the estimated proportion of neonatal deaths (Figure 4).

### 3.8 Risk Factors for Neonatal Mortality

In this systematic review and meta‐analysis, lack of ANC, asphyxia, low‐birth weight, preterm birth, and hypothermia were identified as significant risk factors for neonatal deaths. Neonates born to mothers without ANC follow‐up had 2.7 times higher odds of death compared to those whose mothers received ANC (AOR = 2.7, 95% CI: 1.5–3.96). Similarly, the odds of death were higher among neonates with asphyxia (AOR = 5.1, 95% CI: 3.0–7.1), low‐birth weight (AOR = 4.1, 95% CI: 1.6–6.6), and preterm birth (AOR = 2.2, 95% CI: 1.3–3.1) compared to those without these conditions. Additionally, hypothermic neonates had 2.4 times higher odds of death than those without hypothermia (AOR = 2.4, 95% CI: 1.2–3.7) (Table 4).

**TABLE 4:** Risk factors for neonatal mortality.

Variable	Categories	Number of included studies	Pooled AOR	*I* ^2^, *p* value
Residence	Rural	5	1.3 (95% CI: 0.96–1.6)	—
	Urban		1	
ANC	No	5	2.7 (95% CI: 1.5–3.96)*	—
	Yes		1	
Pregnancy‐related complication	Yes	5	1.9 (95% CI: 0.6–3.1)	19%, 0.294
	No		1	
Asphyxia	Yes	8	5.1 (95% CI: 3.0–7.1)*	55.7%, 0.027
	No		1	
Birth weight	Low	3	4.1 (95% CI: 1.6–6.6)*	—
	Normal		1	
Gestational age	Preterm	5	2.2 (95% CI: 1.3–3.1)*	—
	Term		1	
Hypoglycemia	Yes	4	1.91 (95% CI: 0.67–3.1)	66.1%, 0.031
	No		1	
Hypothermia	Yes	4	2.4 (95% CI: 1.2–3.7)*	—
	No		1	

Abbreviations: ANC, antenatal care; AOR, adjusted odds ratio; CI, confidence interval.

*Statistically significance.

## 4 Discussion

A substantial number of studies on neonatal deaths in Ethiopia met the eligibility criteria for this systematic review and meta‐analysis. Most of the included studies were facility‐based primary studies, with only a few community‐based studies. In total, the studies included 52,853 live‐born neonates, among whom 5129 deaths were reported.

The pooled proportion of neonatal death among admitted neonates was 14.31% (95% CI: 12.59–16.03). This estimate reflects deaths among facility‐based populations, particularly neonates admitted to hospitals, and should not be interpreted as the national neonatal mortality rate. The higher proportion observed in this study compared with global [1] and regional reports [2, 3] may be explained by the nature of the included studies, which largely involve high‐risk neonates receiving care in health facilities. Socioeconomic disparities between countries may also contribute, as evidence suggests that neonates born into wealthier households have improved survival outcomes [78]. The observed proportion is higher than estimates reported from community‐based and population‐level studies, including DHS analyses conducted in East Africa [4] and Ethiopia [5, 6, 7, 8, 9, 11, 12]. This discrepancy can be attributed to differences in study populations and methodologies. DHS‐based studies rely on household surveys and are susceptible to recall and survival bias, as they generally include only surviving mothers. In contrast, facility‐based studies capture more detailed clinical information but primarily include neonates with complications, which may result in higher observed mortality. However, the current estimate is lower than those reported in some previous studies conducted in Ethiopia [10, 14, 15]. For example, studies focusing specifically on preterm neonates [14, 15], who are at higher risk of death, naturally report higher deaths. Additionally, a prior study in eastern Ethiopia [10] reported higher neonatal mortality, which may be explained by its prospective cohort design that allowed more accurate detection of neonatal deaths compared with retrospective or cross‐sectional studies. Moreover, the pooled estimate should be interpreted cautiously because extreme between‐study heterogeneity was observed (*I* ^2^ = 98.93%), indicating substantial variation in death estimates across settings, populations, and study designs. Therefore, the pooled proportion should not be regarded as a single fixed national figure.

The subgroup analysis revealed substantial regional variation in the proportion of neonatal deaths among admitted neonates. The highest proportions were observed in Sidama (17.4%), Amhara (16.7%), Afar (16.4%), and Oromia (16.3%), whereas lower estimates were reported in Addis Ababa (7.5%) and Tigray (9.1%). Relatively high proportions were also observed in SNNP (14.6%), Somalia (14.8%), and Harari (14.4%). These regional differences may reflect disparities in healthcare access, quality of neonatal care, and the availability of specialized neonatal services. Regions with better equipped hospitals and higher levels of urbanization, such as Addis Ababa, tend to report lower facility‐based deaths, whereas peripheral and underserved regions may admit a higher proportion of high‐risk neonates, contributing to higher death estimates.

Consistent with expectations, facility‐based studies reported a substantially higher proportion of neonatal deaths (15.2%) than community‐based studies (3.3%). This difference is likely attributable to the fact that those facility‐based studies predominantly include neonates with complications requiring hospitalization, whereas community‐based studies capture a broader population that includes healthier neonates. Retrospective studies reported a slightly higher proportion of neonatal deaths (14.6%) than prospective studies (13.5%), which may be partly explained by the larger cumulative sample sizes of retrospective studies, enabling the capture of more neonatal deaths. The proportion of neonatal deaths was relatively consistent across publication periods (14.8% for 2016–2019 vs. 14.3% for 2020–2025), suggesting that facility‐based neonatal deaths have remained largely stable over the past decade despite ongoing efforts to improve neonatal care. Studies with smaller sample sizes (≤421) reported a higher proportion of neonatal deaths (16.3%) than larger studies (>421; 13.4%). Meta‐regression analysis confirmed that sample size was a significant predictor of effect size, with larger studies reporting lower proportions of neonatal deaths. This pattern may reflect small‐study effects, whereby smaller studies tend to report higher estimates because of the inclusion of higher‐risk populations, limited representativeness, or random variation.

In this study, lack of ANC, birth asphyxia, low‐birth weight (LBW), preterm birth, and hypothermia were identified as significant risk factors for neonatal deaths. These findings are consistent with evidence from other low‐income settings, where infections, asphyxia, and prematurity are the leading causes of neonatal death [79]. Neonates born to mothers without ANC follow‐up had a higher risk of death, highlighting the importance of improving ANC coverage and quality. Previous studies in Ethiopia have also reported suboptimal ANC utilization [80], which may contribute to adverse neonatal outcomes [15, 16]. Birth asphyxia was strongly associated with neonatal deaths. This may be due to pregnancy‐related complications [81, 82] and inadequate intrapartum care, which can lead to oxygen deprivation and subsequent organ damage [83]. Similarly, preterm birth and low‐birth weight were significant factors of neonatal deaths, consistent with findings from previous studies [15, 16, 18, 19]. These conditions are associated with multiple complications, including respiratory distress [20, 21], feeding difficulties [17], and increased susceptibility to infection [84, 85]. Hypothermia was also identified as a significant risk factor, aligning with existing evidence [20, 21] and emphasizing the importance of early thermal care and appropriate neonatal management.

### 4.1 Strengths and Limitations

This study synthesized evidence from multiple district‐level primary studies across different regions of Ethiopia, resulting in a large sample size and improved precision of estimates. Most regions were represented, enhancing the overall coverage of the analysis. Subgroup analyses were conducted to explore sources of heterogeneity, including study design, region, and publication year. In addition, publication bias was assessed and adjusted using trim‐and‐fill methods.

However, several limitations should be considered. First, most included studies were facility‐based, which limits the generalizability of the findings to the broader population. The estimates primarily reflect mortality among admitted neonates rather than population‐level neonatal mortality. Second, the use of secondary data in some studies may have resulted in incomplete records and limited availability of important variables, restricting the ability to control for confounding factors. Third, substantial heterogeneity was observed across studies, likely due to differences in study design, population characteristics, and healthcare settings. Fourth, EDHS studies were excluded due to methodological differences, which limit national representativeness. After adjusting for publication bias using trim‐and‐fill analysis, the pooled estimate decreased substantially, suggesting possible overestimation in the original analysis. Finally, variation in the definitions and measurement of risk factors across studies may affect comparability.

## 5 Conclusion and Policy Implications

### 5.1 Conclusion

A substantial proportion of neonatal deaths occurred among neonates admitted to healthcare facilities in Ethiopia, indicating a considerable burden of mortality within facility settings. The major factors associated with neonatal death were lack of ANC, birth asphyxia, low‐birth weight, preterm birth, and hypothermia. These findings underscore the need to strengthen maternal and neonatal healthcare services across the continuum of care, with particular emphasis on improving the quality of facility‐based care, early identification of high‐risk pregnancies, and timely management of neonatal complications. Because the available evidence was derived predominantly from health‐facility‐based studies, the findings primarily reflect mortality among admitted neonates receiving care in healthcare facilities rather than the overall neonatal population in Ethiopia. Therefore, the results should be interpreted with caution and may not be generalizable to all neonates at the national level.

### 5.2 Implications for Policy and Practice

The high proportion of neonatal deaths observed in facility‐based settings underscores the need for targeted interventions to improve the quality of neonatal care. Policymakers and healthcare providers should focus on strengthening ANC services, improving intrapartum care to prevent birth asphyxia, and enhancing the management of preterm and low‐birth‐weight neonates. Early identification and management of hypothermia should also be prioritized. Efforts should be directed toward improving the quality of care in neonatal intensive care units and ensuring the availability of essential resources and trained healthcare professionals. In addition, strategies should be developed to enhance the continuum of care from pregnancy through the postnatal period. Future research should include more community‐based studies to provide nationally representative estimates and a more comprehensive understanding of neonatal mortality in Ethiopia.

## Author Contributions

Temesgen Gebeyehu Wondemeneh and Oumer Abdulkadir Ebrahim came up with the idea and were fully involved in the identification, article review, data extraction, quality assessment, analysis, draft writing, and manuscript revision. Temesgen Gebeyehu Wondemeneh was heavily involved in the analysis, draft preparation, and revision of the manuscript. The final version of the manuscript to be considered for publication was read and approved by Temesgen Gebeyehu Wondemeneh and Oumer Abdulkadir Ebrahim. Temesgen Gebeyehu Wondemeneh and Oumer Abdulkadir Ebrahim also agreed to share equal responsibility for all aspects of this research project.

## Funding

The authors have nothing to report.

## Ethics Statement

The authors have nothing to report.

## Consent

The authors have nothing to report.

## Conflicts of Interest

The authors declare no conflicts of interest.

## Supporting information




**Supporting File 1:** Checklist of Preferred Reporting Items For Systematic Reviews and Meta‐Analysis (PRISMA 2020).

**Supporting File 2:** Comprehensive search strategy for neonatal mortality.

**Supporting File 3:** Extracted data for neonatal mortality.

**Supporting File 4:** The quality of included studies.

## Data Availability

All data generated or analyzed during this study are included in this article.
