# Supplementary material for: Neonatal Mortality and Its Associated Factors Among Neonates in Ethiopia: A Systematic Review and Meta‐Analysis
Source: Public Health Chall. 2026 Aug 10;5(3):e70332. doi: 10.1002/puh2.70332 (PMC13455547; doi:10.1002/puh2.70332)
Supplement: Supplementary file 2 [file PUH2-5-e70332-s002.docx]

S2 File. Comprehensive search strategy for neonatal mortality in Ethiopia

| Databases | Key search terms or phrases | |
| --- | --- | --- |
| **Web of science** | (“neonatal mortality” OR “neonatal death” OR “newborn death”) AND Ethiopia | |
| Limited by | research article | |
| Total articles | 113 | |
| **Scopus** | (“neonatal mortality” OR “neonatal death” OR “newborn death”) AND Ethiopia | |
| Limited by | research articles and open access and publication year between 2015 and 2026 | |
| Total articles | 210 | |
| **PubMed** | (“neonatal mortality” OR “neonatal death” OR “newborn death”) AND Ethiopia | |
| Limited by | free full text and publication year between 2015 and 2026 | |
| Total articles | 428 | |
| **Google scholar** | allintitle: (“neonatal mortality” OR “neonatal death” OR “newborn death”) AND Ethiopia | |
| Limited by | publication year between 2015 and 2026 | |
| Total articles | 139 | |
| **African Journals Online** | (“neonatal mortality” OR “neonatal death” OR “newborn death”) AND Ethiopia | |
| Total articles | 329 | |
| **Total articles retrieved from all** **databases** | | 1219 |
