# Supplementary material for: Neonatal Mortality and Its Associated Factors Among Neonates in Ethiopia: A Systematic Review and Meta‐Analysis
Source: Public Health Chall. 2026 Aug 10;5(3):e70332. doi: 10.1002/puh2.70332 (PMC13455547; doi:10.1002/puh2.70332)
Supplement: Supplementary file 3 [file PUH2-5-e70332-s003.docx]

| **ID** | **Authors, publication years** | **Region** | **Study design** | **Study subjects** | **Sample Size** | **Case (%)** | **prevalence** |
| --- | --- | --- | --- | --- | --- | --- | --- |
| 1. | Deressa A, et al. (33), 2019 | Sidama | Retrospective | Neonates | 325 | 111 | 34.20% |
| 2. | Tadesse AW, et al.(34), 2021 | Afar | Retrospective | Neonates | 391 | 57 | 14.60% |
| 3. | Demisse AG, et. al (35),2017 | Amhara | Retrospective | Neonate | 769 | 110 | 14.30% |
| 4. | Farah AE, et al.(36), 2018 | Somali | Retrospective | Neonates | 792 | 45 | 5.70% |
| 5. | Mersha A, et al.(32), 2019 | SNNP | Prospective | Neonates | 6769 | 65 | 0.96% |
| 6. | Eyeberu A, et al.(37), 2021 | Harar | Retrospective | Neonates | 821 | 118 | 14.40% |
| 7. | Limaso AA, et al.(38), 2020 | Sidama | Prospective | Neonates | 584 | 24 | 4.10% |
| 8. | Yeshaneh A, et al.(39),2021 | Amhara | Prospective | Neonates | 422 | 129 | 30.60% |
| 9. | Alebel A, et al.(40), 2020 | Amhara | Prospective | Neonates | 513 | 109 | 21.30% |
| 10. | Dessie AM, et al.(41), 2023 | Amhara | Retrospective | Neonates | 926 | 41 | 4.40% |
| 11. | Kassie A, et al.(42), 2023 | Amhara | Retrospective | Neonates | 870 | 190 | 21.80% |
| 12. | Mengistu BA, et al.(43), 2020 | Amhara | Prospective | Neonates | 612 | 114 | 18.60% |

S3 File Extracted data for neonatal mortality in Ethiopia

| 13. | | Berhanu B, et al.(44), 2021 | | SNNP | | Retrospective | | Neonates | | 367 | | 39 | | 10.60% | |
| --- | --- | --- | --- | --- | --- | --- | --- | --- | --- | --- | --- | --- | --- | --- | --- |
| 14. | | Chekole B, et al.(45), 2023 | | SNNP | | Retrospective | | Neonates | | 375 | | 85 | | 22.70% | |
| 15. | | Roro EM, et al.(46), 2019 | | Oromia | | Retrospective | | Neonates | | 2090 | | 183 | | 8.80% | |
| 16. | | Hadgu FB, et al.(47), 2020 | | Tigray | | Retrospective | | Neonates | | 1785 | | 298 | | 16.70% | |
| 17 | | Thomas G, et al.(48), 2022 | | Dire Dawa | | Retrospective | | Neonates | | 376 | | 43 | | 11.40% | |
| 18 | | Getahun GK, et al.(49), 2024 | | Addis Ababa | | Retrospective | | Neonates | | 459 | | 14 | | 3.10% | |
| 19 | | Chan GJ, et al.(50), 2024 | | Amhara | | Retrospective | | Neonates | | 2411 | | 75 | | 3.10% | |
| 20 | | Mohamed HA, et al.(51), 2022 | | Somali | | Retrospective | | Neonates | | 510 | | 95 | | 18.60% | |
| 21 | | Mengesha HG, et al.(52), 2017 | | Tigray | | Prospective | | Neonates | | 1152 | | 68 | | 5.90% | |
| 22 | | Taye K, et al.(30), 2023 | | Sidama | | Retrospective | | Neonates | | 225 | | 32 | | 14.20% | |
| 23. | Kokeb M, et al.(53), 2016 | | Amhara | | Retrospective | | Neonates | | 325 | | 75 | | 32.10% | |  |
| 24. | Gebremichael MW, et al. (31), 2023 | | Tigray | | Retrospective | | Neonates | | 11 306 | | 189 | | 1.67% | |  |
| 25. | Ayichew MZ, et al.(54), 2022 | | Addis Ababa | | Retrospective | | Neonates | | 570 | | 44 | | 7.70% | |  |
| 26. | Osman MO, et al.(55), 2020 | | Somali | | Retrospective | | Neonates | | 385 | | 79 | | 20.50% | |  |
| 27. | Samuel D, et al.(56), 2019 | | SNNP | | Retrospective | | Neonates | | 332 | | 67 | | 20.20% | |  |
| 28. | Demis SK, et al.(57),2020 | | Amhara | | Retrospective | | Neonates | | 422 | | 52 | | 12.30% | |  |
| 29. | Tolossa T, et al.(58), 2022 | | Oromia | | Prospective | | Neonates | | 412 | | 63 | | 15.30% | |  |
| 30. | Derese T, et al.(59), 2020 | | Dire Dawa | | Retrospective | | Neonates | | 499 | | 44 | | 8.80% | |  |
| 31. | Abera T, et al.(60), 2021 | | Oromia | | Retrospective | | Neonates | | 289 | | 53 | | 18.34% | |  |

| 32. | Mekonnen T, et al.(61),2018 | SNNP | Retrospective | Neonates | 1316 | 300 | 22.80% |
| --- | --- | --- | --- | --- | --- | --- | --- |
| 33. | Tewabe T, et al.(62), 2018 | Amhara | Retrospective | Neonates | 391 | 52 | 13.29% |
| 34. | Orsido TT, et al.(63), 2019 | SNNP | Retrospective | Neonates | 964 | 159 | 16.50% |
| 35. | Menalu MM, et al.(64), 2022 | Addis Ababa | Retrospective | Neonates | 434 | 48 | 11.10% |
| 36. | Gudayu TW, et al. (65), 2020 | Amhara | Retrospective | Neonates | 504 | 87 | 17.30% |
| 37. | Moges S, et al.(66), 2021 | SNNP | Retrospective | Neonates | 1104 | 207 | 18.80% |
| 38. | Cavallin F, et al. (67), 2020 | Oromia | Retrospective | Neonates | 4282 | 709 | 17.00% |
| 39 | Awoke A B, et al.(68), 2025 | Amhara | Retrospective | Neonates | 323 | 28 | 8.7% |
| 40 | Simeneh GT, et al.(69), 2025 | Addis Ababa | Retrospective | Neonates | 313 | 27 | 8.6% |
| 41 | Zekarias W, et al.(70),2024 | Afar | Retrospective | Neonates | 479 | 87 | 18.16% |
| 42 | Enawgaw AS, et al.(71), 2025 | Amhara | Retrospective | Neonates | 638 | 217 | 34% |
| 43 | Adugna B, et al.(72), 2025 | Amhara | Retrospective | Neonates | 1598 | 163 | 10.2% |
| 44 | Jemal M, et al.(73), 2024 | SNNP | Prospective | Neonates | 1080 | 56 | 5.2% |
| 45 | Feyissa GD, et al.(74), 2026 | Oromia | Prospective | Neonates | 504 | 119 | 23.6% |
| 46 | Haile GB, et al.(75), 2025 | Tigray | Prospective | Neonates | 334 | 42 | 12.6% |
| 47 | Fentaw N, et al.(76), 2024 | Amhara | Retrospective | Neonates | 604 | 117 | 19.4% |

SNNP=Southern Nation Nationality People
